# Genome-Wide Characterization of the Phosphate Starvation Response in *Schizosaccharomyces pombe*

**DOI:** 10.1186/1471-2164-13-697

**Published:** 2012-12-12

**Authors:** Ian Carter-O’Connell, Michael T Peel, Dennis D Wykoff, Erin K O’Shea

**Affiliations:** 1 Howard Hughes Medical Institute, Faculty of Arts and Sciences, Center for Systems Biology, Northwest Labs, Harvard University, 52 Oxford Street, Cambridge, MA, 02138, USA; 2 Department of Molecular and Cellular Biology, Harvard University, Faculty of Arts and Sciences, Center for Systems Biology, Northwest Labs, 52 Oxford Street, Cambridge, MA, 02138, USA; 3 Department of Chemistry and Chemical Biology, Harvard University, Faculty of Arts and Sciences, Center for Systems Biology, Northwest Labs, 52 Oxford Street, Cambridge, MA, 02138, USA; 4 Department of Biology, Villanova University, 800 Lancaster Ave, Villanova, PA, 19085, USA

**Keywords:** Phosphate starvation pathway, *pho7*^*+*^, *csk1*^*+*^, *S. pombe* chIp-seq, Gene expression

## Abstract

**Background:**

Inorganic phosphate is an essential nutrient required by organisms for growth. During phosphate starvation, *Saccharomyces cerevisiae* activates the phosphate signal transduction (PHO) pathway, leading to expression of the secreted acid phosphatase, *PHO5*. The fission yeast, *Schizosaccharomyces pombe*, regulates expression of the *ScPHO5* homolog (*pho1*^*+*^) via a non-orthologous PHO pathway involving genetically identified positive (*pho7*^*+*^) and negative (*csk1*^+^) regulators. The genes induced by phosphate limitation and the molecular mechanism by which *pho7*^*+*^ and *csk1*^+^ function are unknown. Here we use a combination of molecular biology, expression microarrays, and chromatin immunoprecipitation coupled with high-throughput sequencing (ChIP-Seq) to characterize the role of *pho7*^*+*^ and *csk1*^*+*^ in the PHO response.

**Results:**

We define the set of genes that comprise the initial response to phosphate starvation in *S. pombe*. We identify a conserved PHO response that contains the *ScPHO5* (*pho1*^*+*^), *ScPHO84* (SPBC8E4.01c), and *ScGIT1* (SPBC1271.09) orthologs. We identify members of the Pho7 regulon and characterize Pho7 binding in response to phosphate-limitation and Csk1 activity. We demonstrate that activation of *pho1*^*+*^ requires Pho7 binding to a UAS in the *pho1*^*+*^ promoter and that Csk1 repression does not regulate Pho7 enrichment. Further, we find that Pho7-dependent activation is not limited to phosphate-starvation, as additional environmental stress response pathways require *pho7*^*+*^ for maximal induction.

**Conclusions:**

We provide a global analysis of the transcriptional response to phosphate limitation in *S. pombe*. Our results elucidate the conserved core regulon induced in response to phosphate starvation in this ascomycete distantly related to *S. cerevisiae* and provide a better understanding of flexibility in environmental stress response networks.

## Background

Inorganic phosphate (Pi) is an essential nutrient required for signal transduction, energy metabolism, and biochemistry in all organisms. Maintaining a constant, stable concentration of internal inorganic phosphate is a major challenge for biological systems. Because external concentrations of inorganic phosphate fluctuate unpredictably, microorganisms have evolved strategies to sense external phosphate levels [1-3], communicate this information to the nucleus [4,5], and induce transcription to respond to phosphate flux [6-8]. The phosphate signal transduction (PHO) pathway in the budding yeast, *Saccharomyces cerevisiae*, is the most thoroughly studied example of phosphate homeostasis in eukaryotes [9-11].

The transcription factors Pho4 and Pho2 play a key role in the phosphate starvation response in *S. cerevisiae*. When cells are grown in conditions where inorganic phosphate is plentiful, Pho4 is multiply phosphorylated by the cyclin-dependent kinase-cyclin (CDK-cyclin) complex, Pho85-Pho80 [12]. When Pho4 is phosphorylated, it is localized to the cytoplasm [13,14], does not interact with Pho2 [15], and the PHO regulon is not expressed. During phosphate starvation, the CDK inhibitor Pho81 binds to the secondary metabolite *myo*-D-inositol heptakisphosphate (IP_7_) and inhibits the Pho85-Pho80 complex [16,17]. Inhibition of Pho85-Pho80 allows Pho4 to be dephosphorylated, enter the nucleus [18], co-operate with Pho2 [7], and induce a set of genes responsible for harvesting inorganic phosphate from the environment [6]. Pho4 function can be conveniently monitored by measuring the activity of the secreted acid phosphatase, Pho5, which is one of the most highly induced members of the PHO response [19,20]. The genes that comprise the PHO regulon have been well characterized and the precise sites within the genome where Pho4 binds during phosphate starvation are known [7,21]. Pho4 regulation occurs in response to changes in external phosphate levels and Pho4 activity is not thought to be regulated by other stress responses.

In this study we ask the following: is the PHO transcriptional response observed in *S. cerevisiae* conserved in the distantly related ascomycete, *Schizosaccharomyces pombe*? *S. pombe* presents an interesting opportunity for addressing this question because: (1) *S. pombe* did not experience a recent whole-genome duplication event – thought to contribute to specialization [22] – possibly preventing the PHO response from developing a dedicated regulatory network; (2) the orthologs for the PHO pathway either do not exist (*PHO81, PHO2, PHO4*) or are not involved in the PHO response (*PHO80, PHO85*) in *S. pombe*[23]; and (3) recent work utilizing a deletion collection in *S. pombe* has outlined a basic regulatory structure for the Pi-inducible, secreted acid phosphatase *pho1*^*+*^ (the ortholog to *PHO5*) creating an opportunity for comparison with the *S. cerevisiae* PHO response [24]. During phosphate starvation *S. pombe* Pho7 – a putative transcription factor containing a Zn_2_Cys_6_ binuclear cluster [25] – activates *pho1*^*+*^ expression. Csk1 – a CDK-activating kinase-activating kinase (CAKAK) [26] – represses *pho1*^*+*^ expression in high-Pi conditions. Epistasis analysis indicates that Pho7 acts downstream of Csk1.

In this study, we explore how these factors affect transcriptional output by characterizing the PHO transcriptional response in *S. pombe*. We analyze this response as a function of phosphate, Pho7, and Csk1 availability using DNA microarrays. We delineate a core PHO transcriptional response comprising the genes *pho1*^*+*^, SPBC8E4.01c (an *S. cerevisiae PHO84* ortholog), and SPBC1271.09 (an *S. cerevisiae GIT1* ortholog), whose induction in response to phosphate starvation is conserved between *S. cerevisiae* and *S. pombe*. Interestingly, while these three genes share a functionally analogous regulatory pathway (i.e. activation through a transcription factor that is normally repressed by a kinase) we find that the mechanism for regulation differs widely between species. Our analysis of the Pho7-regulated transcriptional output – coupled with a global profile of Pho7 binding to promoters of stress responsive genes – leads us to the conclusion that, unlike Pho4, Pho7 plays a role in multiple stress response pathways. We conclude that while there is a core PHO transcriptional response shared between these two ascomycetes, the systems logic and specialization of PHO components varies widely.

## Results and discussion

### *pho7*^*+*^ and *csk1*^*+*^ regulate a core subset of the PHO response in *S. Pombe*

The kinetics and maximal output of transcription vary widely between different environmental stress response pathways [27-29]. To outline the specific PHO response in *S. pombe* for subsequent analysis, and to avoid indirect activation of non-phosphate starvation regulated genes, we performed a single time-dependent, genome-wide expression analysis of wild-type *S. pombe* cells in medium lacking inorganic phosphate (no-Pi conditions, see Additional file 1).

The starvation time-course revealed two distinct responses to phosphate starvation. The rapid response contained 63 genes that exhibited an increase in expression at 120 minutes post-starvation (red lines in Additional file 1, genes listed in Additional file 2; see Methods for gene selection criteria). This class contains the secreted acid-phosphatase, *pho1*^*+*^ (orthologous to *ScPHO5*), SPBC8E4.01c (orthologous to *ScPHO84*), and SPBC1271.09 (orthologous to *ScGIT1*). As *pho1*^*+*^ and SPBC8E4.01c induction has been previously observed in response to Pi starvation [24,30], we believe that this set accurately reflects the genes that respond rapidly to changes in external Pi. In contrast, the slower response (86 additional genes, blue lines in Additional file 1, genes listed in Additional file 2; see Methods for gene selection criteria) was significantly enriched for genes previously implicated in a generalized stress response [27]. We focused our attention on the fast responding genes to avoid indirect effects caused by persistent stress in cells.

Previous work indicated that *pho7*^*+*^ and *csk1*^*+*^ are important regulators of *pho1*^*+*^ expression [24]; we expected that they would also play a significant role in regulating additional components of the PHO response. To test our hypothesis, we probed the transcriptional profiles of wild-type, *pho7*Δ, *csk1*Δ, and *pho7*Δ*csk1*Δ strains in high-Pi and no-Pi conditions at 120 minutes post-starvation using DNA hybridization microarrays.

**Figure 1:**
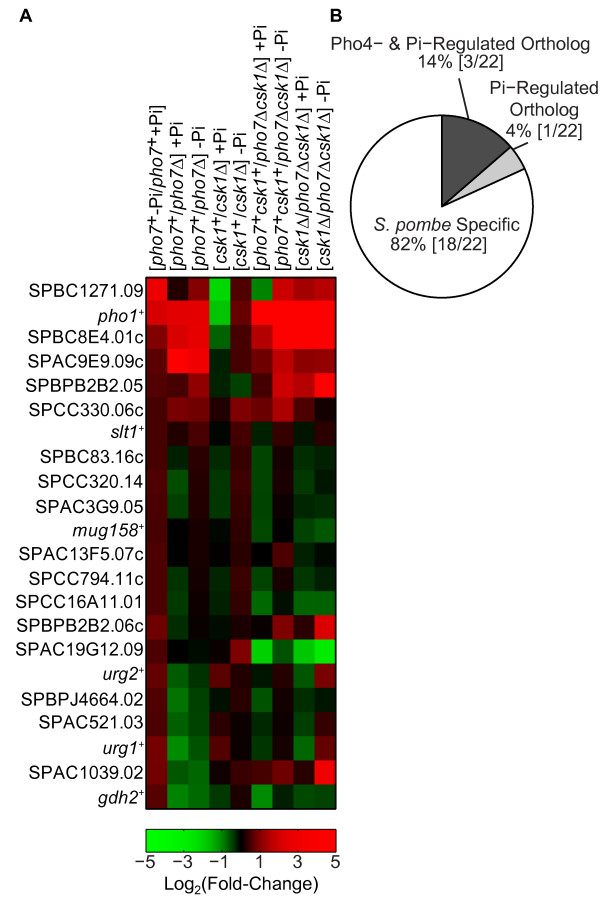
**Pho7 and Csk1 regulate a core set of genes induced in response to phosphate limitation. **(**A**) Heat map showing the fold change (log_2_ scale) of Pi-starvation induced genes in a wild-type background (first column), a *pho7*Δ strain in high- and no-Pi conditions vs. a *pho7*^*+*^ strain (second and third), a *csk1*Δ strain in high- and no-Pi conditions vs. a *csk1*^*+*^ strain (fourth and fifth), a *pho7*Δ*csk1*Δ strain in high- and no-Pi conditions vs. a *pho7*^*+*^*csk1*^*+*^ strain (sixth and seventh), and a *csk1*Δ strain in high- and no-Pi conditions vs. a *pho7*Δ*csk1*Δ strain (eighth and ninth) as measured by microarray analysis. Thresholds used for gene selection are described in the materials and methods. For a full list of Pi, *pho7*^*+*^, and *csk1*^*+*^ dependent genes see Additional file 3. (**B**) Pie chart showing the percentage of genes induced in response to Pi limitation with Pi- and Pho4-dependent orthologs in *S. cerevisiae*[7].

We identified 22 genes induced in response to Pi limitation (Figure 1A, first column, applying a p-value cutoff of 0.10; see Methods for gene selection criteria). If expression of these genes is dependent on the activity of *pho7*^*+*^, then their transcript abundance should decrease in a *pho7*Δ strain when compared to a wild-type background (both starved of Pi). Induction of 31.8% (7/22) of these genes during Pi starvation is *pho7*^*+*^ dependent (Figure 1A, third column). If the repressor (*csk1*^*+*^) prevents this induction by *pho7*^*+*^ in high-Pi conditions, then transcript abundance in a *csk1*Δ strain compared to the *csk1*^*+*^ background in high-Pi conditions should increase. Three genes display this response (Figure 1A, fourth column). A complete listing of all genes regulated by Pi, *pho7*^*+*^, and/or *csk1*^*+*^, along with their orthologs in *S. cerevisiae* can be found in Additional file 3. Finally, comparisons of the *pho7*Δ, *csk1*Δ, and *pho7*Δ*csk1*Δ double deletion strains confirm the previously described epistatic relationship between *pho7*^*+*^ and *csk1*^*+*^ (i.e. a loss of *pho7*^*+*^ in a *csk1*Δ switches constitutive expression to uninducible expression) (Figure 1A, last two columns).

When phosphate is limiting, *S. cerevisiae* Pho4, along with Pho2, induces the transcription of genes required for phosphate acquisition [7]. The orthologs of Pho4, Pho2, Pho81, and Pho80 are not found in *S. pombe* or involved in the PHO response, raising the question: does a functionally analogous signaling pathway involving *pho7*^*+*^ and *csk1*^*+*^ regulate the PHO transcriptional response in *S. pombe*? Comparing the Pho4, Pi-starvation induced genes in *S. cerevisiae*[7] with the *pho7*^*+*^, Pi-starvation induced genes in *S. pombe* reveals an overlap of only three orthologs (Figure 1B). For *pho1*^*+*^, SPBC8E4.01c, and SPBC1271.09 a similar system of transcription factor activation, with repression by a kinase in high-Pi conditions, occurs. Unlike Pho85-Pho80 regulation of Pho4 in *S. cerevisiae*, most of the *pho7*^*+*^-mediated response is independent of *csk1*^*+*^ regulation. Further, a large segment (82%) of the observed *S. pombe* PHO response is not conserved between *S. pombe* and *S. cerevisiae*. Therefore, the primary role for *pho7*^*+*^ and *csk1*^*+*^ in the PHO pathway is regulating the core set of phosphate harvesting and transport genes (*pho1*^*+*^, SPBC8E4.01c*,* and SPBC1271.09).

### Pho7 is enriched at the PHO core promoters during Pi starvation

Pho7 is classified as a putative transcription factor because it possesses a Zn_2_Cys_6_ binuclear cluster (ZC), a DNA binding domain for a number of transcription factors [31,32]. To test if Pho7 binds to *pho7*^*+*^-regulated promoters, cells containing a functional, epitope tagged version of Pho7 (Pho7-TAP) were grown in high-Pi or no-Pi medium and purified DNA associated with Pho7 was processed via high-throughput sequencing (ChIP-Seq, see Methods).

Surprisingly, there is widespread Pho7 binding even in high-Pi conditions (1676 peaks out of 4208 passed peak-ID thresholds; see Methods) (Additional file 4). During Pi starvation 367 Pho7-bound sites exhibit an increase in Pho7 enrichment. The highest levels of enrichment were observed in the promoters of Pho7-regulated genes identified in the microarray analysis (for a complete list of the identified peaks, see Additional file 5). Further, there is a distinct overlap between genes whose expression levels are regulated by Pi and/or *pho7*^*+*^ and those whose promoters display enrichment in Pho7 binding. 13 of the 22 Pi dependent genes (p-value = 8.1e-8), 16 of the 29 *pho7*^*+*^ dependent genes (p-value = 9.5e-9), and 6 of the 7 genes regulated by both Pi and *pho7*^*+*^ (p-value = 1.3e-5) have promoters that are enriched for Pho7 binding in no-Pi medium (p-values were determined using a hypergeometric test, see Methods for details). These results are very different from the global binding profile of Pho4 in *S. cerevisiae*. In that system, Pho4 is only recruited to the promoters of PHO regulated genes during phosphate starvation and, even then, only to relatively few locations (115) within the genome [7].

**Figure 2:**
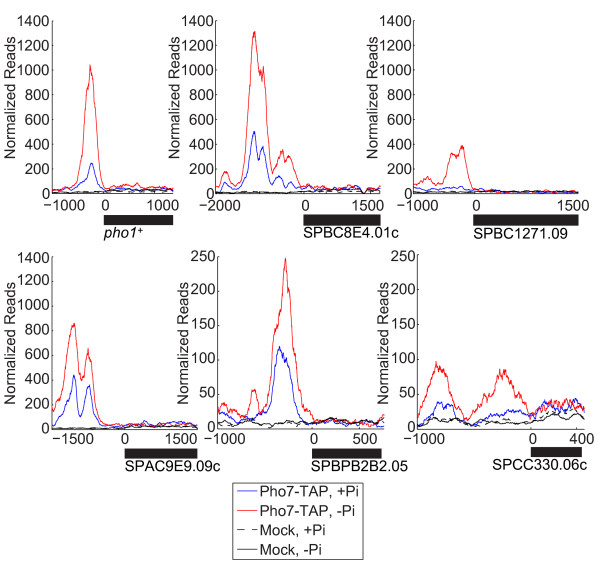
**Pho7 is recruited to phosphate-regulated promoters during Pi starvation.** Cells lacking the TAP epitope tag (Mock) or containing an epitope fused version of Pho7 (Pho7-TAP) were assayed for their enrichment of Pho7 at the *pho1*^*+*^ promoter via ChIP-Seq. Cells were either incubated in high-Pi conditions (blue) or starved (red) for 2 hours before cross-linking. Reads were normalized to total counts for each chromosome. Magnified ChIP-Seq profiles for *pho1*^*+*^, SPBC8E4.01c, SPBC1271.09, SPAC9E9.09c, SPBPB2B.05, and SPCC330.06c are shown. Mock samples lacking the TAP epitope (black) were treated identically to experimental samples. Coordinates are relative to the initial ATG, with gene products identified by black bars.

Pho7 binding is significantly enriched in the promoters of *pho1*^*+*^, SPBC8E4.01c, SPBC1271.09, SPAC9E9.09C, SPBPB2B2.05, and SPCC330.06c in no-Pi conditions (Figure 2). In the case of *pho1*^*+*^, SPBC8E4.01c, SPAC9E9.09C, and SPBPB2B2.05 there is enrichment of Pho7 even in high-Pi conditions (when compared to mock treated samples). This explains the previously noted basal expression of *pho1*^*+*^ in *S. pombe*[30,33] – it appears that Pho7 is bound and activating transcription even in the absence of stress (Figure 1A, second column). Based on our microarray results, we note that increased Pho7 enrichment during Pi starvation is not due to an increase in *pho7*^*+*^ transcript abundance (0.05 log_2_ fold-change in no-Pi vs. high-Pi conditions). Moreover, we observe no significant difference in Pho7-TAP protein levels during Pi starvation (Additional file 6). Together these observations suggest that the enrichment of Pho7 in the promoters of Pho7-regulated genes is attributable to increased affinity for the promoter and not due to an increase in Pho7 abundance. Further, the number of distinct Pho7 binding events varies between different promoters (Figure 2), and we do not observe a clear correlation between the number of binding events and transcriptional up-regulation. Our attempts to identify a DNA binding motif for Pho7 based solely on our ChIP-Seq data were unsuccessful (data not shown).

### Csk1 does not regulate Pho7 promoter occupancy

**Figure 3:**
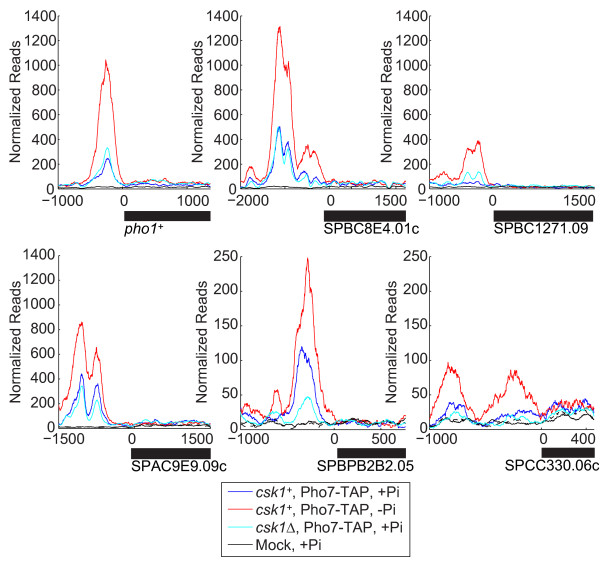
**Csk1 does not regulate Pho7 Promoter enrichment.** ChIP-Seq profiles for *pho1*^*+*^, SPBC8E4.01c, SPBC1271.09, SPAC9E9.09c, SPBPB2B.05, and SPCC330.06c are shown. Cells from either a *csk1*^*+*^ (blue) or *csk1*Δ (cyan) background were incubated in high-Pi conditions for 2 hours. Mock samples lacking the TAP epitope (black) were treated identically to experimental samples. Coordinates are relative to the initial ATG, with gene products identified by black bars. Reads were normalized as previously described.

Motivated by the following observations, we studied what effect loss of Csk1 would have on Pho7 enrichment in high-Pi conditions: (1) Csk1 represses the expression of the core PHO genes (*pho1*^*+*^, SPBC8E4.01c, and SPBC1271.09) in high-Pi medium (Figure 1B, column 4); and (2) a decrease in Pi results in enrichment of Pho7 at the core PHO promoters (Figure 2). If binding of Pho7 to the PHO promoters is necessary to drive increased transcriptional output, and Csk1 represses transcription by preventing Pho7 binding, then a loss of Csk1 in high-Pi conditions should result in an increase in Pho7 binding (mimicking the no-Pi binding profile observed for Pho7 in wild-type cells). Pho7 binding at the *pho1*^*+*^ promoter in a *csk1*Δ background is mildly increased compared to a *csk1*^*+*^ background (Figure 3). This increase in binding is less than the observed increase in Pho7 binding in *csk1*^*+*^ cells grown in no- vs. high-Pi conditions (1.2-fold versus 4-fold), suggesting that Csk1 is not the major regulator of Pho7 binding at the *pho1*^*+*^ promoter.

We then examined the global effect of Csk1 loss on the binding profile of Pho7 using ChIP-Seq with *csk1*Δ cells grown in high-Pi conditions. Unlike the enrichment during Pi starvation, deletion of Csk1 does not result in a global increase in Pho7 binding in high-Pi conditions (Additional file 7). At the core PHO responsive genes we observe either no change (SPBC8E4.01c) or a slight increase in Pho7 binding in the *csk1*Δ strain (*pho1*^*+*^ and SPBC1271.09), which is still well below the enrichment seen during Pi starvation (Figure 3). As we observed during Pi starvation, the loss of Csk1 does not influence either *pho7*^*+*^ transcript abundance (-0.12 log_2_ fold-change, [*csk1*Δ vs. *csk1*^+^] +Pi) or Pho7-TAP protein levels (Additional file 6). We draw two conclusions from these data: (1) the level of Pho7 bound in high-Pi conditions would be sufficient to induce high levels of transcription if not for the repressive action of Csk1; and (2) Csk1 does not repress Pho7 activity by preventing Pho7 from binding to the promoters of responsive genes.

### A Pho7 upstream activating sequence (UAS) and an independent Pi sensing module control *pho1*^*+*^ expression

**Figure 4:**
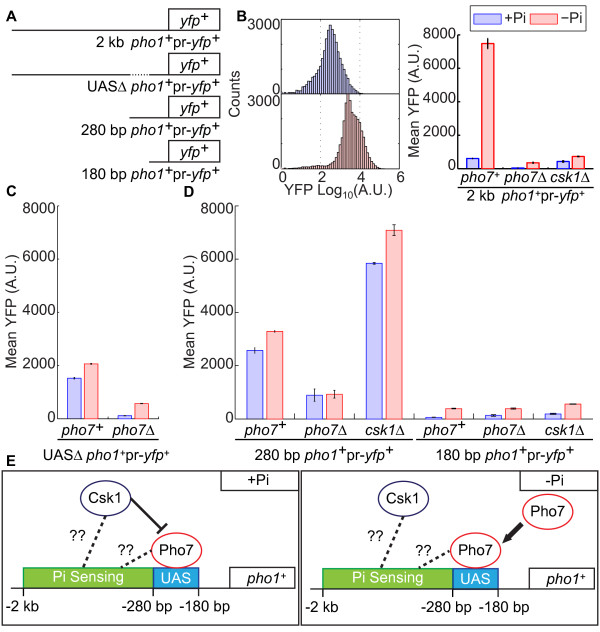
**A Pho7 UAS in the *pho1***^***+***^**promoter is necessary expression. **(**A**) Schematic of *pho1*^*+*^ promoter variants used for expression analysis. 2 kb *pho1*^*+*^pr-*yfp*^*+*^: full promoter, 280 bp *pho1*^*+*^pr-*yfp*^*+*^: promoter trimmed to only the Pho7-UAS, 180 bp *pho1*^*+*^pr-*yfp*^*+*^: promoter trimmed beyond the Pho7-UAS, UASΔ *pho1*^*+*^pr-*yfp*^*+*^: full promoter lacking only the 20 bp under the Pho7 ChIP-Seq peak. See text for vector construction. (**B**) FACS histogram profile for the 2 kb *pho1*^*+*^pr-*yfp*^*+*^ construct transformed into a *pho7*^*+*^ strain. Cells were grown in either high-Pi (blue) or no-Pi (pink) media for 4 hours prior to fixation and counting. Bar graph depicting mean YFP intensity for the 2 kb *pho1*^*+*^pr-*yfp*^*+*^ construct transformed into a *pho7*^*+*^, *pho7*Δ, and *csk1*Δ strain and grown in either high-Pi (blue) or no-Pi (pink) media are given as the average of three biological replicates ± SE. (**C**) The UASΔ *pho1*^*+*^pr-*yfp*^*+*^ construct was transformed into *pho7*^*+*^ or *pho7*Δ strains and treated as in B. (**D**) The noted constructs were transformed into *pho7*^*+*^, *pho7*Δ, or *csk1*Δ backgrounds and treated as in B. (**E**) Model detailing the role of Csk1 in repressing Pho7 function at the *pho1*^*+*^ promoter. In high-Pi conditions Pho7 is not recruited to the UAS and Csk1 interacts near the UAS to prevent full Pho7 activity. When the external concentration of Pi drops, additional Pho7 is recruited to the promoter and Csk1 repression is relieved resulting in maximal induction. Proper regulation is controlled within a Pi sensing module located between bases -2000 and -280 in an unknown manner.

Based on our ChIP-Seq results, we know Pho7 binds between nucleotides -280 and -180 in the *pho1*^*+*^ promoter. To determine whether the sequences in this region are necessary and/or sufficient for Pho7-dependent, Pi-starvation induced expression, we utilized an *in vivo* strategy for confirming Pho7-promoter interactions using exogenous expression plasmids. Briefly, differing lengths of the *pho1*^*+*^ promoter driving the expression of yellow fluorescent protein (*yfp*^*+*^) were constructed in a vector and transformed into *pho7*^*+*^, *pho7*Δ, and *csk1*Δ backgrounds (Figure 4A). *yfp*^*+*^ expression was measured using FACS with mean YFP intensity serving as a proxy for promoter activity (see Methods). The 2 kb segment of the *pho1*^*+*^ promoter activates *yfp*^+^ expression during Pi-starvation - it exhibits a ~7-fold increase in YFP intensity upon starvation (Figure 4B). This induction is dependent on Pho7, as it is abolished in a *pho7*Δ background (Figure 4B). Surprisingly, we find that loss of Csk1 does not result in high-levels of *yfp*^*+*^ expression with the 2 kb reporter in *csk1*Δ cells grown in either high-Pi or no-Pi growth media. While the levels of expression are above those seen in *pho7*^*+*^ cells grown in high-Pi conditions and *pho7*Δ cells grown in any condition, they are significantly lower than the levels observed in a *pho7*^*+*^ background in no-Pi conditions (Figure 4B). Based on our evidence that trimming the *pho1*^*+*^ promoter beyond 1 kb results in high level, constitutive expression of *yfp*^*+*^ in *csk1*Δ cells (Figure 4D, discussed below), our results with the 2 kb fragment in the *csk1*Δ background might be complicated by: (1) an additional repressor element located between 2 kb and 280 bp in the *pho1*^*+*^ promoter; and/or (2) differences between the chromatin structure/promoter architecture of the endogenous *pho1*^*+*^ locus and the exogenous 2 kb *pho1*^*+*^pr-*yfp*^+^ vector that are influencing transcription in the *csk1*Δ cells. Therefore, we focused on the behavior of the shorter construct (280 bp *pho1*^*+*^pr-*yfp*^+^; Figure 4D) as a proxy for the interaction of Csk1 at the region bound by Pho7-TAP in the *pho1*^+^ promoter.

If the *pho1*^*+*^ promoter sequence bound by Pho7-TAP in the ChIP-Seq experiment is necessary for activation of *pho1*^*+*^ transcription during Pi-starvation, then deletion of this region should result in a loss of *yfp*^*+*^ expression during Pi-starvation. To test this hypothesis, we generated a construct in which the 20 bp centered under the Pho7-TAP ChIP-Seq signal were deleted (Figure 4A, UASΔ *pho1*^+^pr-*yfp*^*+*^). In high-Pi growth conditions, the loss of the Pho7 bound region results in a slight increase in *yfp*^+^ expression (Figure 4C, compare blue columns to the blue columns in Figure 4B), and in Pi-starvation this UASΔ *pho1*^+^pr-*yfp*^*+*^ construct no longer fully activates *yfp*^*+*^ expression (Figure 4C, compare column 2 to column 2 in Figure 4B). The loss of *pho7*^*+*^ results in a further decrease in expression from this UASΔ *pho1*^+^pr-*yfp*^*+*^ construct (Figure 4C, column 4). It is possible that Pho7 recognizes additional segments of the promoter, though such contributions to activation in Pi-limiting conditions are modest. Together these results demonstrate that the Pho7-TAP bound promoter element is necessary for Pho7-dependent transcriptional activation during Pi-limitation. We have termed this region the Pho7 upstream activating sequence (UAS).

To test whether the Pho7 UAS is sufficient for Pho7-dependent, Pi-limitation induced transcriptional activation we deleted all but the first 280 bp of the *pho1*^+^ promoter and assayed *in vivo yfp*^*+*^ expression (Figure 4A, 280 bp *pho1*^+^pr-*yfp*^+^). *yfp*^*+*^ expression from the 280 bp *pho1*^*+*^pr-*yfp*^*+*^ construct is elevated in high-Pi conditions, and is only marginally activated during Pi-starvation (Figure 4D, *pho7*^*+*^, column 1). Expression from this construct is reduced in a *pho7*Δ background and is unaffected by Pi limitation – thus, expression in high-Pi conditions and the modest expression increase in Pi-limiting conditions are dependent on Pho7 (Figure 4D, *pho7*Δ, column 2). In each background tested, the mean YFP intensities from the 280 bp *pho1*^*+*^pr-*yfp*^*+*^ construct vary by less than 1.5 fold between high-Pi and no-Pi conditions (Figure 4D, left panel). This is in contrast to both *yfp*^*+*^ expression from the 2 kb *pho1*^*+*^pr-*yfp*^+^ construct and endogenous expression of *pho1*^*+*^ during Pi-starvation which exhibit >10-fold induction in Pi limitation. Thus, the Pho7 UAS is necessary but not sufficient for Pho7-dependent transcriptional activation during Pi-starvation.

Interestingly, the 280 bp *pho1*^*+*^pr-*yfp*^*+*^ construct is capable of inducing full *yfp*^*+*^ expression in no-Pi conditions in a *csk1*Δ background (Figure 4D, *csk1*Δ, column 3). However, the 280 bp *pho1*^*+*^pr-*yfp*^*+*^ construct is not capable of relieving Csk1 repression in Pi-starvation conditions – expression is not induced in response to Pi limitation. Trimming beyond the Pho7 UAS results in transcriptionally inactive promoters in all backgrounds tested (Figure 4D, columns 4-6, 180 bp *pho1*^*+*^pr-*yfp*^*+*^). We conclude that there must be promoter elements present in the region between -2 kb and -280 bp in the *pho1*^*+*^ promoter that act as a Pi-sensor: (1) preventing partial Pho7-dependent activation in high-Pi conditions; and (2) are important for Csk1 de-repression during Pi-starvation.

Our FACS and ChIP-Seq results lead us to the following model for Pho7 and Csk1 regulation at the *pho1*^*+*^ promoter. In high-Pi conditions some Pho7 is bound to the UAS in the *pho1*^*+*^ promoter. Pho7 in this state drives basal expression of *pho1*^*+*^. Csk1, through an interaction with either Pho7 or elements near the UAS (directly or indirectly), prevents the full activation of *pho1*^*+*^ expression. The upstream Pi-sensor in the promoter ensures that Csk1 remains repressive in these conditions through an as yet unspecified mechanism. During Pi starvation, Csk1 repression is relieved and additional Pho7 is recruited to the *pho1*^*+*^ promoter, driving maximal expression (Figure 4E). Investigating the promoter elements and transcription factors that comprise the Pi-sensor – as well as the use of this promoter structure at additional Pho7-dependent and Pi-starvation inducible promoters – is an exciting area for future research.

### Pho7 regulates gene expression in response to multiple stress conditions

During our expression analysis we noticed a set of genes with decreased expression in high phosphate conditions in a *pho7*Δ background that are not induced during phosphate starvation (Additional file 3). Additionally, Pho7 is bound to the promoters of a number of these genes in the ChIP-Seq analysis (Additional file 8). These observations raise the following question: is Pho7 dedicated solely to the phosphate starvation pathway, like Pho4, or does it play a broader role in the stress response?

**Figure 5:**
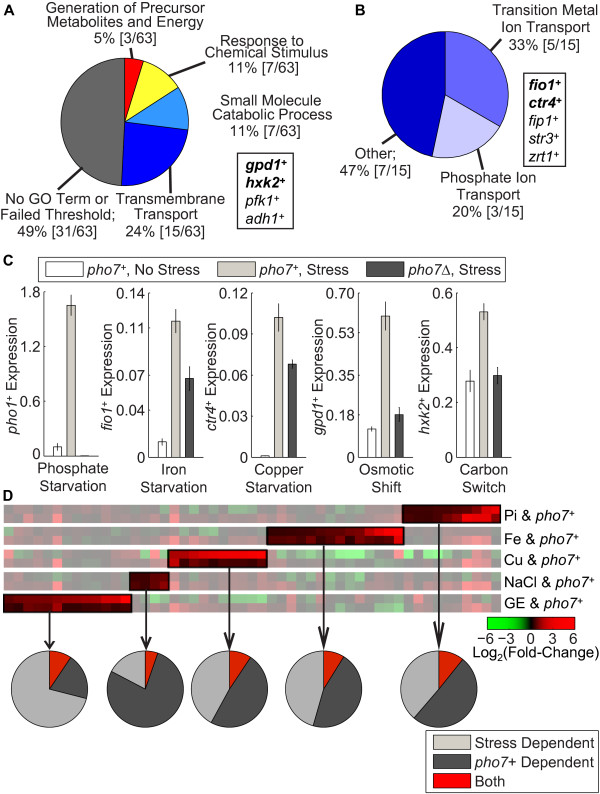
***pho7***^***+***^**functions in additional stress responses. **(**A**) Genes with a Pi- and *pho7*^*+*^-component based on microarray analysis were cross-referenced with a list of genes with *pho7*^*+*^ enrichment within 800 bp of the initial ATG (ChIP-Seq). The pie chart shows the GO Term enrichment for the identified genes as compared to a background model of all *S. pombe* transcripts. The boxed inset highlights a number of genes that were found in the small molecule catabolic process class, with those in bold serving as the initial targets for expression analysis. P-values for the enrichment against the background model are given. (**B**) The transmembrane transport category from A was expanded to identify specific ions whose transport is regulated by *pho7*^*+*^. Bold candidate genes served as initial target for expression analysis. (**C**) *pho7*^*+*^ cells were incubated in either normal (white) or stress (light gray) conditions, and the expression of the appropriate gene was measured by RT-qPCR. *pho7*Δ cells were also stressed (dark grey) to identify the level of *pho7*^*+*^-dependent regulation. Expression was normalized to *act1*^*+*^. Shown is the average of three biological replicates ± SE. (**D**) Heat map displaying the fold-change (log_2_ scale) for genes induced during stress in a *pho7*^*+*^-dependent manner. The top track in each sub-column compares expression in replete versus stress conditions for wild-type cells; the bottom track compares *pho7*^*+*^ to *pho7*Δ cells in the displayed stress (Pi: phosphate starvation, Fe: iron starvation, Cu: copper starvation, NaCl: osmotic shift, GE: carbon switch). Boxed regions identify genes that are induced in response to stress and require *pho7*^*+*^ for their stress response. Pie charts depict the percentage of genes responding to stress in a *pho7*^*+*^ independent manner (light grey), repressed by the loss of *pho7*^*+*^ but unaffected by stress (dark grey), and dependent on both (red).

To answer this question, we identified the genes from our microarray analysis that display an increase in expression between the wild-type and *pho7*Δ backgrounds (in either +Pi or -Pi conditions; thresholds described in materials and methods) and asked whether their promoters contained a significant peak of bound Pho7 within 800 bp of the start codon – there are 63 genes that meet these requirements. This gene set was then processed through the Gene Ontology Tools: Term Enrichment algorithm [34], allowing us to look for biological functions over-represented in our gene set. For a GO term to be considered enriched, we required at least three unique gene products be included in the term family and the enrichment must have a p-value ≤ 0.01. 32 of the original 63 genes meet these requirements and are classified by the highest parent term available (Figure 5A). 15 of these 32 genes are involved in transmembrane transport, including 3 involved in phosphate ion transport. Characterizing the transmembrane category in further depth we find genes involved in iron, copper, and zinc transport (Figure 5B). A complete listing of GO Terms and the enriched gene set can be found in Additional file 9.

From the 32 identified genes, we utilized *gpd1*^*+*^, *hxk2*^*+*^, *fio1*^*+*^, and *ctr4*^*+*^ expression as proxies for Pho7-mediated transcriptional induction in various stress conditions. Gpd1 is a glycerol-3-phosphate dehydrogenase that synthesizes glycerol and is essential for survival during osmotic stress [35]. During osmotic stress, glycerol pools increase, protecting the cell. Hxk2 is a hexokinase that plays a role in regulating alternative carbon utilization when glucose sources are limited [36]. It is maximally induced in response to a switch from glucose to glycerol as a carbon source. Fio1, in conjunction with Fip1, comprises the oxidase-permease iron transport system responsible for harvesting iron in depleted conditions [37]. During iron repletion *fio1*^*+*^ is repressed by the activity of Fep1, an iron sensing transcription factor [38]. During iron starvation *fio1*^*+*^ is de-repressed and induced ~70-fold. Finally, Ctr4 is a high-affinity copper transporter that is induced in copper depletion conditions by Cuf1, a copper sensing transcription factor [39]. We designed RT-qPCR primer sets for each of these genes and measured their expression as a function of osmotic, iron, copper, and carbon utilization stress in both *pho7*^*+*^ and *pho7*Δ backgrounds (see Methods).

As previously demonstrated [24], loss of Pho7 completely abrogates induction of *pho1*^*+*^ in no-Pi medium (Figure 5C, first panel). For each of the additional stresses tested, the loss of Pho7 causes a significant decrease (p-value ≤ 0.05) in the maximal induction of the target gene (Figure 5C). The Pho7 dependence of these genes varies, with some (*fio1*^*+*^, *ctr4*^*+*^) showing a relatively minor Pho7 component, while others (*gpd1*^*+*^, *hxk2*^*+*^) appear fully dependent on Pho7 to reach a peak level of induction in stress. None of the Pho7-regulated responses were as dramatic as that observed for Pi starvation, which may indicate that Pho7 plays a more subtle role in coordinating expression at Pi-independent loci. Given this subtle response, the only recent availability of a deletion collection for *S. pombe*[40], and the fact that Pho7 function was unverified until recently, it is not surprising that the more general role for Pho7 has not been previously observed.

It remains possible that the stress response effects we observe are artifacts limited to only the few genes we studied using RT-qPCR. Using microarray analysis with RNA collected from *pho7*^*+*^ or *pho7Δ* cells grown in non-stress and stress conditions, we examined the stress response mediated by *pho7*^*+*^ in the above conditions. We find that of 274 genes induced in the stress conditions studied, 44 genes are *pho7*^*+*^ dependent (7 genes are induced in multiple stress conditions) (Figure 5D and Additional file 10). *pho7*^*+*^ is responsible for coordinating between 11.7-23.5% of the total stress response in each condition (for comparison, *pho7*^*+*^ coordinates 21.7% of the Pi starvation response using these thresholds). In each stress we find enrichment of distinct GO terms. The set of iron-responsive, *pho7*^*+*^-regulated genes is significantly enriched for the biological process of iron assimilation (*fio1*^*+*^, *fip1*^*+*^, *str3*^*+*^, *sib2*^*+*^, p-value = 5.2e-09), the set of copper-responsive, *pho7*^*+*^-regulated genes is enriched for copper ion transport (*ctr4*^*+*^ and *ctr5*^*+*^, p-value = 5.6e-05), and the osmotic shock-responsive, *pho7*^*+*^-regulated gene set is enriched for metal ion transport (*zrt1*^*+*^ and *ctr4*^*+*^, p-value = 7.8e-04). With the carbon switch, *pho7*^*+*^-regulated response we see an enrichment of genes responsible for small molecule metabolism as well as conjugation. The reasoning for the conjugation process enrichment or why *pho7*^*+*^ would be involved is unclear. Overall, the biological processes are generally linked with transmembrane transport, suggesting that *pho7*^*+*^ is responsible for coordinating the correct transport of nutrients required for each stress response. Unlike the system in *S. cerevisiae*, where the central Pi-starvation regulator is tightly linked with the PHO response, the *pho7*^*+*^ based system in *S. pombe* functions differentially in a number of stress response networks.

## Conclusions

In this study we have defined and characterized the gene regulatory network in *S. pombe* responsible for coordinating the response to inorganic phosphate starvation. There are two distinct temporal responses in the PHO pathway in *S. pombe*: a fast response concerned with immediately harvesting inorganic phosphate from the environment and transporting it into the cell, and a slower one associated with a general stress response. Within the fast response we define a core PHO regulon comprised of the *pho1*^*+*^, SPBC8E4.01c, and SPBC1271.09 genes whose induction in response to phosphate starvation, and regulatory behavior, has been conserved between *S. pombe* and *S. cerevisiae*.

**Figure 6:**
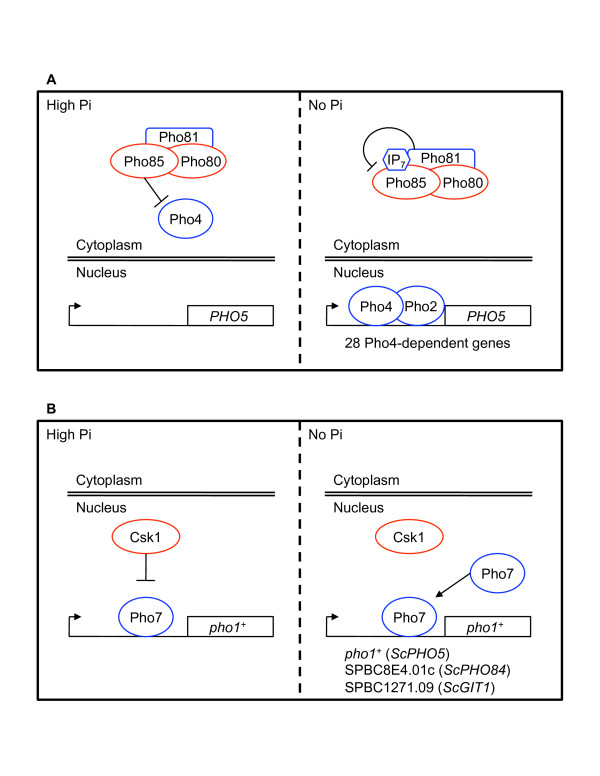
**A comparison of the *S. cerevisiae *and *S.pombe *PHO signaling pathways.** (**A**) A schematic depicting the PHO signaling pathway in *S. cerevisiae*. In conditions where Pi is plentiful (high Pi, left) Pho4 is multiply phosphorylated by the Pho85-Pho80 complex and is excluded from the nucleus. Depletion of inorganic phosphate (no Pi, right) leads to the IP_7_-Pho81-dependent inhibition of Pho85-Pho80. Relief of Pho85-Pho80 mediated repression allows unphosphorylated Pho4 to enter the nucleus, interact with Pho2 at PHO-specific genes, and induce transcription. (**B**) A schematic depicting the PHO signaling pathway in *S. pombe*. In high Pi conditions (left) some Pho7 is bound to the promoters of Pi- and *pho7*^*+*^-dependent genes. Bound Pho7 is prevented from reaching maximal activation by the repressive effects of Csk1. Loss of external phosphate (right) results in the recruitment of additional Pho7 to the promoter and the relief of Csk1-mediated repression. The Pho7-dependent genes identified in this study that are regulated in this manner are listed (*S. cerevisiae* orthologs in parenthesis).

Unlike the PHO response in *S. cerevisiae*, however, the positive regulator in *S. pombe* is bound to the *pho1*^*+*^ and SPBC8E4.01c promoters irrespective of external phosphate availability. Using our Pho7-TAP ChIP-Seq dataset, we identified a single Pho7 binding region in the *pho1*^*+*^ promoter. This region – located between nucleotides -265 and -245 – serves as a Pho7-dependent UAS, which is necessary for transcriptional activation of *pho1*^*+*^. The interaction of Pho7 with the UAS leads to a basal expression of the secreted acid phosphatase in high-Pi conditions. The 1.8 kb region preceding the UAS in the *pho1*^*+*^ promoter is required for Pi sensing, coordinating the activation by Pho7 and repression by Csk1 based on Pi availability. Csk1 prevents full activation of Pho7 during phosphate replete conditions and repression is maintained even in the minimal UAS construct (280 bp *pho1*^*+*^pr-*yfp*^*+*^). During phosphate starvation this inhibition is relieved (through an unknown mechanism) and additional Pho7 is recruited to a number of sites throughout the genome, causing further induction of *pho7*^*+*^-dependent genes (see Figure 6 for a comparison of the *S. cerevisiae* and *S. pombe* PHO pathways). In previous work, Csk1 was shown to regulate transcription by activating the positive transcription elongation factor b (P-TEFb) ortholog, Cdk9 [41]. Cdk9 coordinates transcript elongation and processing, and its full activation by Csk1 leads to an increase in CTD kinase activity [42]. How this generally positive regulatory network is switched to an inhibitory role in the PHO system remains an open question.

We were also surprised to find that Pho7 was bound throughout the genome in both high-Pi and no-Pi conditions. We had thought based on previous evidence that Pho7, like Pho4, would be specific to the PHO response. Instead we demonstrate that Pho7 binds within the promoters of additional stress responsive genes and plays a role in iron, copper, osmotic, and alternative carbon utilization stress. Each stressor elicits a different *pho7*^+^-dependent transcriptional response, though it appears that the main regulatory role of *pho7*^*+*^ is coordinating stress-specific transmembrane transport. There must exist some mechanism to either direct Pho7 to the proper location for inducing the correct genes or activate Pho7 at only the appropriate locations (or some mixture of both). In *S. cerevisiae*, the osmotic, oxidative, and glucose limitation stress responses are mediated by the transcription factor Msn2 [43]. In normal conditions, Msn2 is phosphorylated and its entry into the nucleus is limited [44]. Different stresses elicit distinct dynamics of nuclear transport, leading to different transcriptional outputs [29]. Given that Pho7 is bound to the genome constitutively, we do not expect that nuclear exclusion will play as large a role as it does with Msn2 regulation, but it remains possible that differential post-translational modifications are responsible for this combinatorial activation by Pho7. Pho7 may be playing a more passive role in regulation, with additional factors determining Pho7 genomic localization.

Nonetheless, we have demonstrated that within the evolutionary parallel signal transduction networks that comprise the PHO pathway there exists a core PHO transcriptional regulon. The specific mechanisms involved in regulating the PHO response in *S. cerevisiae* and *S. pombe* show remarkable flexibility. An interesting area for future research centers on the environmental factors that contributed to the development of these two parallel networks. Why is the PHO response in *S. pombe* under the control of a general stress transcription factor, Pho7, while *S. cerevisiae* has developed the phosphate starvation specific pathway for Pho4 activation? What are the environmental pressures that favor a “leaky” response in *S. pombe* and a tightly controlled one in *S. cerevisiae*? Broadly speaking, our study provides a framework for determining the fundamental requirements for regulating phosphate homeostasis in Ascomycota and the specific points in the signal transduction pathway that can be altered as conditions merit.

## Methods

### Growth conditions and strains

*S. pombe* cells were maintained in previously described YES or EMM media [45]. The yeast strains used were: DP1 (972 *h*^*-*^), DP18 (*ura4-D18 ade*^*+*^*leu*^*+*^*h*^*+*^), DP81 (*pho7*Δ*KANMX6* 972 *h*^*-*^), DP94 (Pho7-TAP*KANMX6* 972 *h*^*-*^), DP106 (*csk1*Δ*NATMX6* 972 *h*^*-*^), DP109 (*csk1*Δ*KANMX6 ura4-D18 ade*^*+*^*leu*^*+*^*h*^*+*^), DP111 (*pho7*Δ*KANMX6 ura4-D18 ade*^*+*^*leu*^*+*^*h*^*+*^), DP113 (*pho7*Δ*KANMX6 csk1*Δ*NATMX6* 972 *h*^*-*^), DP114 (Pho7-TAP*KANMX6 csk1*Δ*NATMX6 ura4*^*-*^ 972 *h*^*-*^), and DP115 (Pho7-TAP*KANMX6 csk1*Δ*NATMX6 ura4*^*-*^ 972 *h*^*+*^). The functionality of the Pho7-TAP allele was confirmed by both liquid phosphatase assay and RT-qPCR analysis and it behaves as *pho7*^*+*^. To tag Pho7 and delete *csk1*^*+*^ we utilized a PCR fragment containing the marker of interest flanked by homologous regions for the specific gene target. Cells were transformed with lithium acetate and polyethylene glycol 8000 [45]. Primers used for deletion or tagging are found in Additional file 11. To provide consistency with previously published results for inorganic phosphate starvation, all starvation experiments were conducted with cells incubated in a 90%SD-10%EMM media, which has been previously described [24].

### Microarray analysis and data processing

Strains were grown in 90%SD-10%EMM medium containing 10 mM KH_2_PO_4_ (high-Pi) at 30°C until they reached early-log phase (OD_600_=0.15-0.25). Cells were collected via filtration, washed twice, transferred to fresh media lacking Pi (no-Pi), and grown at 30°C for up to 4 hours. Immediately prior to starvation (t=0), 20 mL of cells were added to 30 mL of methanol kept at -65°C to prevent further transcription or RNA degradation. At 30, 60, 120, and 240 minutes post-starvation this process was repeated. Cells were left in methanol for 10 minutes, pelleted, washed quickly in autoclaved water, re-suspended in 750 uL of RNAlater (Ambion), and snap-frozen in liquid nitrogen. RNA was extracted using the RNeasy Mini kit (Qiagen). cDNA was generated in a reverse transcriptase reaction using 10 μg total RNA with a 1:1 mixture of oligo-dT and random hexamer primers (Operon) and a 2:3 ratio of amino-allyl-dUTP:dTTP (Sigma). Superscript II RT (Invitrogen) was added and the reaction mixture was incubated at 42°C for 2.5 hours. cDNA was purified using a PCR purification kit (Qiagen) after completing hydrolysis of remaining RNA. An equal amount of cDNA from each time point was pooled to provide the reference sample. Purified cDNA samples were labeled using N-hydroxyl succimamide esters of either Cy3 or Cy5 dyes (GE Biosciences). 300 ng of the Cy3 (each individual time point) and 300 ng of the Cy5 (pooled reference) labeled sample was competitively hybridized to custom Agilent 8x15K *S. pombe* two-color expression microarrays (GEO Platform:GPL15827) in 2xGEx Hybridization Buffer (Hi-RPM) (Agilent) for 17 hours at 60°C. Microarrays were washed and immediately scanned using an Axon 4000B scanner [46]. The mean intensity of each spot in the Cy3/Cy5 channels was extracted using the GenePix 5.1 software, followed by lowess and quantile normalization performed with the MATLAB bioinformatics toolbox. Expression ratios for each time point, x, were normalized to t=0 (Log_2_[Cy3_t=x_/Cy5_pool_ – Log_2_[Cy3_t=0_/Cy5_pool_) and thresholds for induced genes were set at ≥ 2σ + median log_2_ fold change for each time point (1.00 log_2_ fold change at 120 minutes, 1.24 log_2_ fold change at 240 minutes). Genes above threshold at both 120 minutes and 240 minutes post-starvation were classified as the rapid response. Genes above only the 240-minute threshold were classified as the slow response. The starvation time course was not repeated. Results for all of the microarray experiments conducted in this study are available through NCBI-GEO [GEO:GSE39478].

To determine the extent of *pho7*^*+*^ and *csk1*^*+*^ regulation within the PHO response we grew the relevant strains (DP1, DP81, DP106, DP113) as described above, with the exception that cells were split into either high-Pi or no-Pi media and grown for 2 hours prior to RNA collection. Two independent biological replicates were performed for each of the conditions tested except for the *pho7*^*+*^*csk1*Δ/*pho7*Δ*csk1*Δ comparison in no-Pi media. For each of these arrays the two probes used to detect each ORF were averaged and treated as single data points with p-values determined using a student’s t-test with a one-tailed distribution against the null hypothesis in the MATLAB software. Thresholds were set at ≥ 1.8 log_2_ fold-change to facilitate comparison with the previously characterized *S. cerevisiae* data set [7]. Genes passing the induction threshold also had to pass a p-value threshold of ≤ 0.10. Clustering analysis was completed using k-means clustering in the Cluster 3.0 program [47] after empirically determining the optimal number of clusters using the MATLAB bioinformatics toolbox.

### Chromatin immunoprecipitation of Pho7-TAP with high-throughput sequencing (ChIP-Seq)

ChIP-Seq was performed on the DP1, DP94, and DP115 strains as previously described [7,48-50]. Cells were grown to early log-phase (OD_600_~0.18) in high-Pi media at 30°C and split into either 200 mL of high-Pi or no-Pi media and grown for 2 hours. Formaldehyde (Sigma) was added to a final concentration of 1% (v/v) to cross-link chromatin, and the reaction was allowed to proceed for 15 minutes. Glycine (Sigma) was then added to a final concentration of 125 mM and incubated for 5 minutes to quench cross-linking. Cells were lysed by bead beating (6 × 2 min on, 2 min off) and chromatin was sheared to 300-600 bp fragments using a Misonix Sonicator 3000. Immunoprecipitation was performed with 100 uL of Protein G Dynabeads (Invitrogen) coupled to 4 uL of anti-Protein A antibody (Sigma). Protein concentrations were measured using a Bradford Assay (Bio-Rad). Following the generation of ChIP lysate three aliquots of 650 μg soluble protein were subject to immunoprecipitation and pooled just prior to elution from the beads. Samples were processed following the Illumina HTS guidelines with libraries of 200-300 bp selected via 2% agarose DNA gels. Libraries were amplified by PCR and purity was determined using an Agilent High-Sensitivity DNA kit on an Agilent Bioanalyzer. Libraries were sequenced on an Illumina HighSeq 2000 and 50bp reads were aligned to the *S. pombe* 972 *h*^*-*^ genome using ELAND. We obtained between 16 million and 54 million reads on average from our samples. Uniquely aligned reads were extended 80 bp from the read start site to cover the average length of insert as determined by the Agilent Bioanalyzer. Results for all of the ChIP-Seq experiments conducted in this study are available through NCBI-GEO [GEO:GSE39498].

To determine which of our enriched regions were actually attributable to a Pho7-TAP binding event we used a modified method from [48]. For each condition analyzed we set a lower threshold for peak discovery equal to the genomic average of reads per base. We set the upper threshold equal to the highest observed read count within the given sample. Using 380 equal increments between these two thresholds we defined peaks that were larger than 100 nucleotides and separated by at least 20 nucleotides. Peaks were compiled at the highest threshold at which they met those standards and full peaks were required to be at least 150 nucleotides distant from the nearest neighbor. Statistical analysis comparing sample enrichment to mock enrichment was performed in MATLAB using previously described methods [48]. Peaks used for subsequent analysis had a ≥ 2-fold enrichment over the genome average and a p-value ≤ 0.005 compared to the mock sample.

To determine the likelihood that the genes determined from microarray analysis to be regulated by Pi and/or *pho7* would also have promoters that contain at least one Pho7 binding site we utilized a hypergeometric test. In this case the p-value is given by:

$$P=1-\sum_{i=0}^{k-1}\frac{\left(\begin{array}{c} M\\ i \end{array}\right)\left(\begin{array}{c} N-M\\ n-i \end{array}\right)}{\left(\begin{array}{c} N\\ i \end{array}\right)}$$

where N is the total number of genes probed by the microarray (5046), M is the number of peaks within -800 and 0 bp of any start codon in no-Pi conditions (570), n is the full set of regulated genes (22 for Pi, 29 for *pho7*^*+*^, and 7 for both), and k is the set of regulated genes with at least one Pho7 peak in the promoter (13 for Pi, 16 for *pho7*^*+*^, and 6 for both).

### Western blotting with Pho7-TAP

DP1, DP94, or DP114 Cells were grown to log-phase in high-Pi media at 30°C. Following collection of a high-Pi sample, cells were washed three times in no-Pi media, transferred to no-Pi media, and grown for either 60 or 120 minutes. Cells were lysed by beadbeating in urea lysis buffer (20 mM Tris-HCl, pH 8.0, 50 mM Na_2_HPO_4_, 8M urea, and 1 mM PMSF). Total protein was quantified on a Nanodrop 2000 (Thermo Scientific) using a BSA standard (Bio-Rad). Equal amounts of total protein for each sample (30 ug) were subjected to separation by SDS-PAGE and transferred to nitrocellulose. Immunoblotting was performed with rabbit IgG (1:1000, Jackson ImmunoResearch) followed by incubation with goat anti-rabbit-HRP (1:5000, Thermo Scientific). Blots were developed using the SuperSignal West Femto Chemiluminescent Substrate (Thermo Scientific) and analyzed on an Alpha Innotech Gel Imagining System.

### *pho1*^*+*^ promoter deletion analysis

Segments of the *pho1*^*+*^ promoter were amplified using PCR and cloned into a *yfp*^*+*^ plasmid using homologous recombination [51] containing the selectable *ura*^*+*^ marker, creating a *pho1*^*+*^pr-*yfp*^*+*^ fusion. Plasmids were transformed into DP18, DP109, or DP111 backgrounds using lithium acetate and polyethylene glycol 8000. Cells were selected based on their ability to grow in EMM-ura media. Cells containing the various plasmids were grown to early log-phase (OD_600_~0.18) in high-Pi media (lacking uracil) at 30°C, collected, washed twice in sterile water, and split into either high-Pi or no-Pi media (both lacking uracil). Cells were grown for 4 hours at 30°C and 100 uL of 10% buffered formalin (Sigma) was added to 900 uL culture. Fixation proceeded for 5 minutes at room temperature prior to washing: once with 0.1M potassium phosphate buffer (pH 8.5) and once with 1.2M sorbitol in 0.1M potassium phosphate buffer (pH 8.5). Cells were resuspended in 1.2M sorbitol, 0.1M potassium phosphate buffer (pH 8.5) and incubated overnight at 4°C.

FACS counting with each sample was performed using a LSR II Analyzer (BD Biosciences). DP18, DP109, and DP111 cells lacking the *yfp*^*+*^ expression system were used to normalize forward-scatter, side-scatter, and autofluorescence for each experiment. 50,000 cells were counted for each experimental condition tested; cells with forward- and side-scatter values between 50,000-150,000 and YFP expression ≥ mean autofluorescence were subject to further analysis. Three biological replicates were performed and the average YFP intensity for the replicates is reported ± SE.

### *pho7*^*+*^ regulation in additional stress response pathways

For each individual stress response, initial cultures of DP1 and DP81 were grown in 90%SD-10%EMM media containing 10 mM KH_2_PO_4_ to early-log phase at 30°C. Cells were collected, washed twice with autoclaved water, and split into the following conditions (all modifications of the high-Pi media): +Pi – 10 mM KH_2_PO_4_, -Pi – 0 mM KH_2_PO_4_, +Fe – 100 uM Fe(III)Cl_3_, -Fe – 250 uM 2-2’-dipyridine (DIP) (Sigma), +Cu – 100 uM Cu(II)SO_4_, -Cu – 100 uM bathocuproine disulphonate (BCS) (Sigma), Osmotic Shift – 1.2M NaCl instead of 0.1M NaCl, and Carbon Switch – 2% glycerol/1% ethanol (GE) instead of 2% glucose (G). Cells were grown for 2 hours and harvested as described above. Recovered RNA was converted into cDNA using the iScript cDNA synthesis kit (Bio-Rad) and subjected to RT-qPCR. Amplification of the *gpd1*^*+*^*, fio1*^*+*^*, ctr4*^*+*^*, hxk2*^*+*^, and *pho1*^*+*^ transcripts were measured for the three independent replicates and transcript abundance was normalized to *act1*^*+*^. Shown is the average ± SE. Primers used in the RT-qPCR analysis can be found in Additional file 11.

Extracted RNA was also subjected to microarray analysis as detailed above. Expression from *pho7*^*+*^ cells in replete conditions was compared to that in stress conditions for each individual stressor to determine the base set of genes that respond in each given stress. The dependence upon *pho7*^*+*^ was determined by comparing the levels of induction in *pho7*^*+*^ cells in stress to induction in *pho7*Δ cells in stress. Based on previous reports [27] the osmotic shift conditions were assayed 20 minutes post-shift to provide a more accurate measure of genes directly induced by osmotic pressure. Extraction of Cy3-Cy5 fluorescence intensity was performed using the GenePix 5.1 software and normalization was completed using the MATLAB bioinformatics toolbox. At least two independent biological replicates were performed for each of the conditions tested. For each of these arrays the two probes used to detect each ORF were averaged and treated as single data points with p-values determined using a student’s t-test with a one-tailed distribution against the null hypothesis in the MATLAB software. Thresholds were set at ≥ 1.5-fold-change for all conditions with the exception of the [-Cu/+Cu] and [GE/G] arrays. For those conditions a significantly larger proportion of genes were induced, so we set the thresholds at ≥ [2σ + median] log_2_ fold-change to ensure a similar sized cohort of analyzed genes. Genes passing the induction threshold also had to pass a p-value threshold of ≤ 0.10.

## Competing interests

The authors declare that they have no competing interests.

## Authors' contributions

The manuscript was written by ICO. Microarrays, ChIP-Seq, and FACS quantization were performed by ICO. DDW provided *S. pombe* strains. MP prepared the *pho1*^*+*^pr-*yfp*^*+*^ constructs. Computational analysis was performed by ICO using modified methods from previously published sources [7,46,48]. EKO and DDW provided supervision throughout and EKO and DDW are the corresponding authors. All authors read and approved the final manuscript.

## Supplementary Material

Additional file 1 **Figure S1.** Temporal Dynamics of the Phosphate Starvation Response in *S. pombe*. (A) Line plot depicting the induction profile for genes displaying a fast (red) or slow (blue) response to phosphate starvation as measured by microarray analysis. Thresholds used to delineate the response time are described in the text. Induction was normalized to the initial sample pre-starvation (t=0). (B) Average expression values for genes in the fast (■) and slow (**O**) response are shown ± SD. Click here for file

Additional file 2 **Table S1. **Microarray Results for Genes Identified in the Pi Starvation Time Course. Genes induced following two hours (fast response, **bold**) or four hours (slow response) of Pi depletion were tabulated and their normalized expression levels (to t=0 min) are shown. Induction thresholds for the fast response were set at ≥ 2σ + median log_2_ fold change for each time point (1.00 log_2_ fold change at 120 minutes, 1.24 log_2_ fold change at 240 minutes). Genes above threshold at both 120 minutes and 240 minutes post-starvation were classified as the rapid response. Genes above only the 240-minute threshold were classified as the slow response. Click here for file

Additional file 3 **Table S2. **Microarray Results for Genes Regulated by Pi Starvation, *pho7*^*+*^, and/or *csk1*^*+*^. Genes induced following Pi starvation, in *pho7*^*+*^ versus *pho7***Δ** cells during Pi starvation, or in *csk1***Δ** versus *csk1*^*+*^ cells in non-stressed (high-Pi) conditions were tabulated and analyzed for orthologs in the *S. cerevisiae* PHO pathway. Induction thresholds were set at ≥ 1.8 fold-change with a p-value ≤ 0.10. Values shown are the average of two independent replicates. Click here for file

Additional file 4 **Figure S2. **Global Pho7-TAP Enrichment During Pi-Starvation. Cells containing the tagged variant of Pho7 (Pho7-TAP) were grown in either high-Pi (blue) or starvation (red) media for 120 minutes prior to cross-linking and ChIP-Seq processing. Shown are the chromosomal enrichment profiles for Pho7 for the *S. pombe* genome. Reads were normalized to total counts for each chromosome. Click here for file

Additional file 5 **Table S3. **Peak List for Pho7-TAP ChIP-Seq Data Set. Pho7-TAP peaks detected in both high-Pi (Hi) or no-Pi (No) conditions with maximum height ≥ 2x genome average are given. Peak detection was performed against a mock sample lacking a TAP epitope as described in materials and methods. Click here for file

Additional file 6 **Figure S3. **Pho7-TAP Protein Levels Remain Constant During Pi Starvation in *csk1*^*+*^ and *csk1***Δ** Backgrounds. *csk1*^*+*^ or *csk1***Δ** cells containing the tagged variant of Pho7 (Pho7-TAP) were grown in high-Pi (+Pi) media followed by Pi starvation for 60 or 120 minutes (-Pi). Western blot analysis reveals similar levels of the Pho7-TAP protein in all conditions as detected by rabbit IgG. The strain lacking the TAP tag is shown (Pho7) as a negative control. The Western analysis was completed in duplicate, shown is a representative blot. Click here for file

Additional file 7 **Figure S4. **Global Pho7-TAP Enrichment in the Absence of Csk1. *csk1*^*+*^ (blue) or *csk1***Δ** (cyan) cells containing the tagged variant of Pho7 (Pho7-TAP) were grown in high-Pi media for 120 minutes prior to cross-linking and ChIP-Seq processing. Shown are the chromosomal enrichment profiles for Pho7 for the *S. pombe* genome. Reads were normalized to total counts for each chromosome. Click here for file

Additional file 8 **Figure S5. **ChIP-Seq Binding Profiles for Pho7-TAP at Non-PHO Promoters. Shown are ChIP-Seq profiles for the genes identified in Figure 6. As previously described, wild-type cells containing Pho7-TAP were grown in either high-Pi (blue) or no-Pi (red) conditions and ChIP-Seq libraries were prepared from purified DNA. For comparison, the ChIP-Seq signal from mock (black) cells grown in no-Pi and *csk1***Δ** (cyan) cells incubated in high-Pi is included. The gene product of interest is plotted based on transcript direction with the plus (+) strand above and the minus (-) strand below. Reads were normalized as described in the text and the location within the genome is plotted on the x-axis. Click here for file

Additional file 9 **Table S4. **Gene Ontology Enrichment for Pho7 Dependent Genes Identified via Microarray and ChIP-Seq Analysis. Genes possessing a *pho7*^*+*^-dependency independent of Pi availability were cross-referenced with the Pho7-TAP peak list to identify *pho7*^*+*^-regulated genes with promoters enriched by Pho7-TAP. Thresholds for induction and peak enrichment are described in materials and methods. The gene set was processed through the AmiGO toolkit [34,52-55] and GO terms containing at least 3 genes with p-values ≤ 0.01 are displayed. Click here for file

Additional file 10 **Table S5. **Microarray Results for *pho7*^*+*^-Dependent Genes Regulated by Phosphate, Iron, Copper, Osmotic, and/or Carbon Switching Stress. *pho7*^*+*^ genes passing the thresholds (described in materials and methods) for each stress condition assayed were tabulated with the corresponding fold-change (Log_2_) and p-values (based on at least two independent biological replicates). Genes regulated in multiple stress conditions by *pho7*^*+*^ are indicated in **bold**. All comparisons between stressed and non-stressed conditions (e.g., [-Pi/+Pi]) were done in a pho7+ background. Genes passing the thresholds for each array condition have fold-change (Log_2_) values indicated in bold. +Pi: 10 mM H2KPO4, -Pi: 0 mM H2KPO4, +Fe: 100 uM Fe(III)Cl3, -Fe: 250 uM DIP, +Cu: 100 uM Cu(II)SO4, -Cu: 100 uM BCS, 1.2M: 1.2M NaCl, 0.1M: 0.1M NaCl, G: 2% glucose, GE: 2% glycerol, 1% ethanol. Click here for file

Additional file 11 **Table S6.** All primers utilized in this manuscript are listed with their primer ID, nucleotide sequence, and experimental purpose. Click here for file
