# Supplementary figures and images for: Genome-Wide Characterization of the Phosphate Starvation Response in Schizosaccharomyces pombe
Source: BMC Genomics. 2012 Dec 12;13:697. doi: 10.1186/1471-2164-13-697 (PMC3556104; doi:10.1186/1471-2164-13-697)

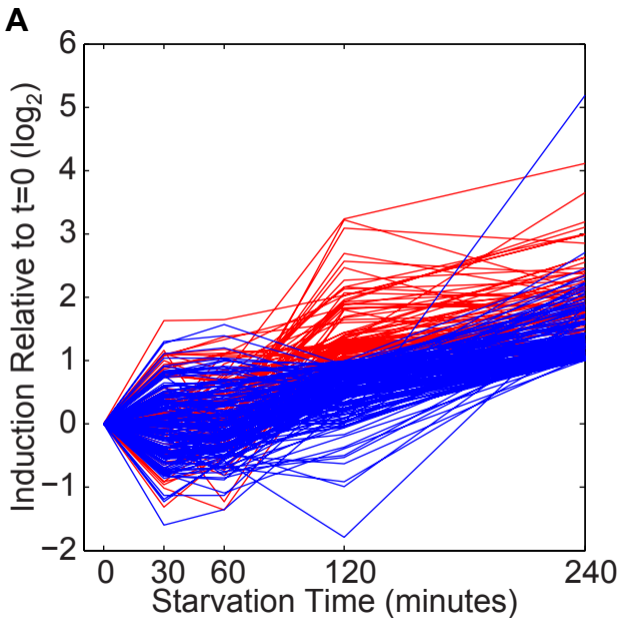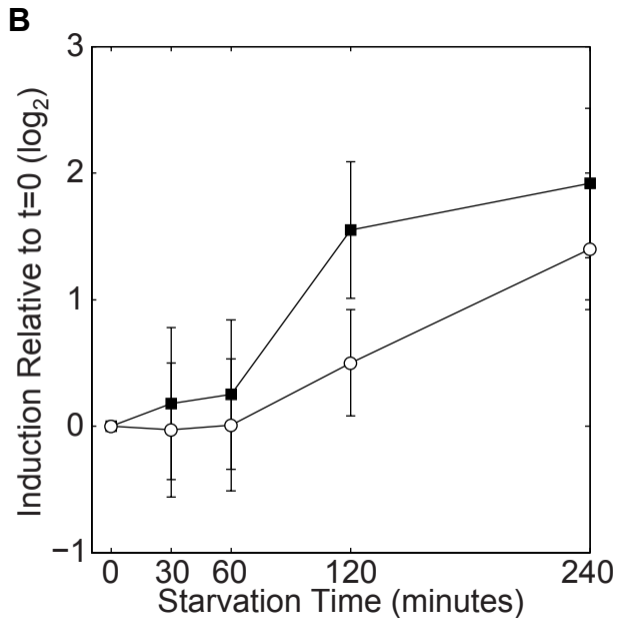

**Figure S1.**

Supplement: Additional file 1 — Figure S1. Temporal Dynamics of the Phosphate Starvation Response in S. pombe. (A) Line plot depicting the induction profile for genes displaying a fast (red) or slow (blue) response to phosphate starvation as measured by microarray analysis. Thresholds used to delineate the response time are described in the text. Induction was normalized to the initial sample pre-starvation (t=0). (B) Average expression values for genes in the fast (■) and slow (O) response are shown ± SD. [file 1471-2164-13-697-S1.pdf]

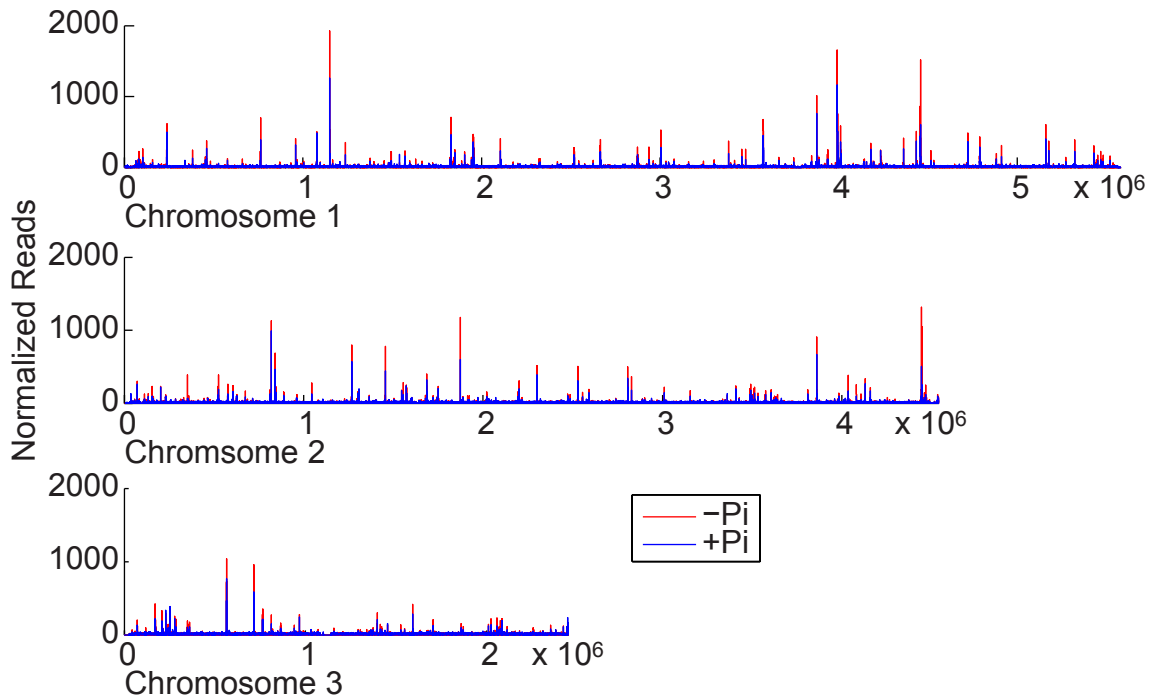

**Figure S2.**

Supplement: Additional file 4 — Figure S2. Global Pho7-TAP Enrichment During Pi-Starvation. Cells containing the tagged variant of Pho7 (Pho7-TAP) were grown in either high-Pi (blue) or starvation (red) media for 120 minutes prior to cross-linking and ChIP-Seq processing. Shown are the chromosomal enrichment profiles for Pho7 for the S. pombe genome. Reads were normalized to total counts for each chromosome. [file 1471-2164-13-697-S4.pdf]

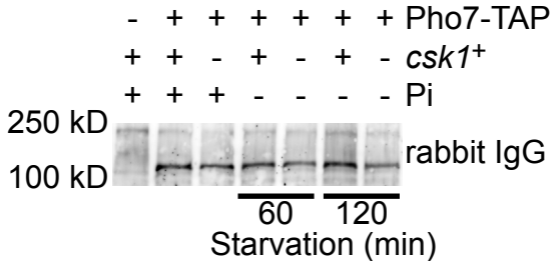

**Figure S3.**

Supplement: Additional file 6 — Figure S3. Pho7-TAP Protein Levels Remain Constant During Pi Starvation in csk1+ and csk1Δ Backgrounds. csk1+ or csk1Δ cells containing the tagged variant of Pho7 (Pho7-TAP) were grown in high-Pi (+Pi) media followed by Pi starvation for 60 or 120 minutes (-Pi). Western blot analysis reveals similar levels of the Pho7-TAP protein in all conditions as detected by rabbit IgG. The strain lacking the TAP tag is shown (Pho7) as a negative control. The Western analysis was completed in duplicate, shown is a representative blot. [file 1471-2164-13-697-S6.pdf]

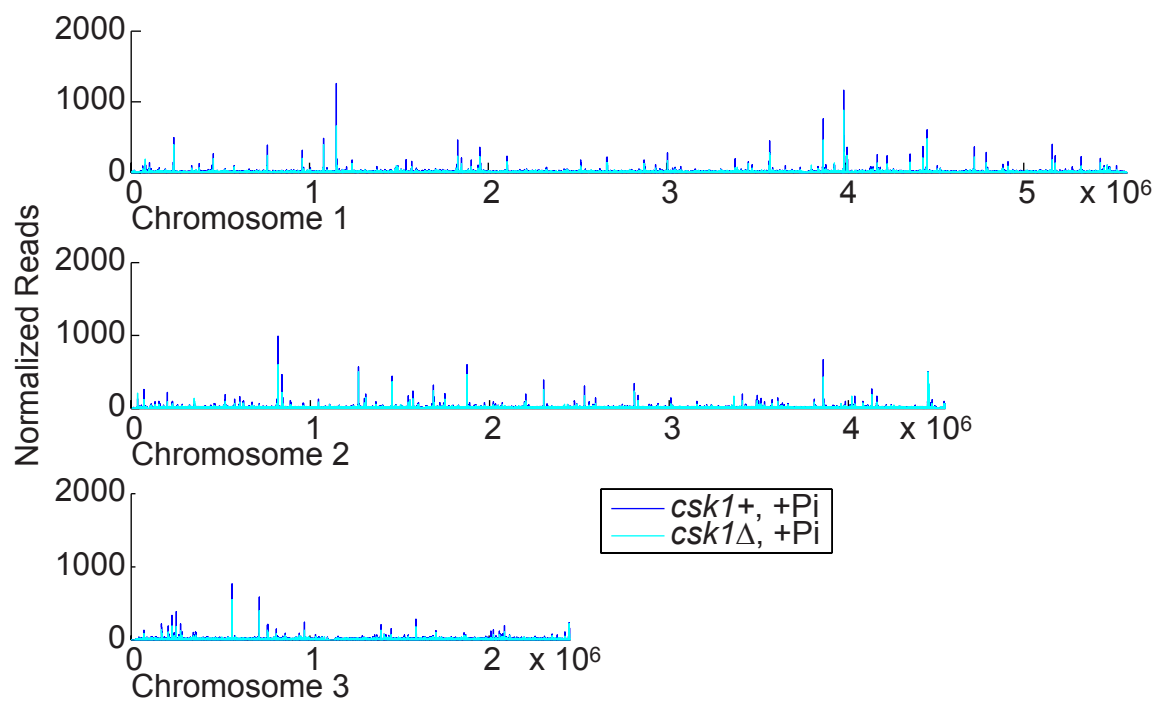

**Figure S4.**

Supplement: Additional file 7 — Figure S4. Global Pho7-TAP Enrichment in the Absence of Csk1. csk1+ (blue) or csk1Δ (cyan) cells containing the tagged variant of Pho7 (Pho7-TAP) were grown in high-Pi media for 120 minutes prior to cross-linking and ChIP-Seq processing. Shown are the chromosomal enrichment profiles for Pho7 for the S. pombe genome. Reads were normalized to total counts for each chromosome. [file 1471-2164-13-697-S7.pdf]

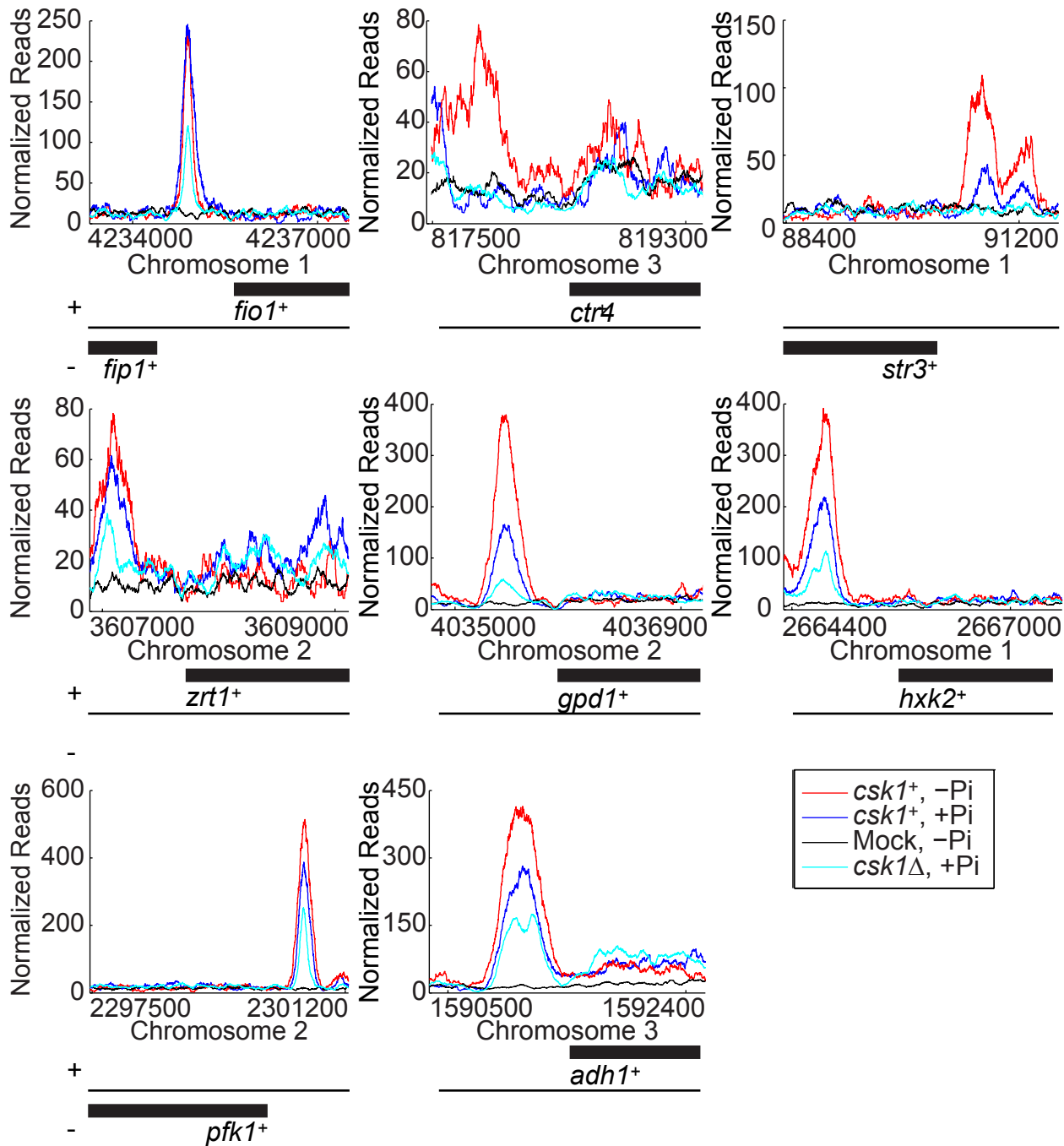

**Figure S5.**

Supplement: Additional file 8 — Figure S5. ChIP-Seq Binding Profiles for Pho7-TAP at Non-PHO Promoters. Shown are ChIP-Seq profiles for the genes identified in Figure 6. As previously described, wild-type cells containing Pho7-TAP were grown in either high-Pi (blue) or no-Pi (red) conditions and ChIP-Seq libraries were prepared from purified DNA. For comparison, the ChIP-Seq signal from mock (black) cells grown in no-Pi and csk1Δ (cyan) cells incubated in high-Pi is included. The gene product of interest is plotted based on transcript direction with the plus (+) strand above and the minus (-) strand below. Reads were normalized as described in the text and the location within the genome is plotted on the x-axis. [file 1471-2164-13-697-S8.pdf]
